# Moss Extracts as Natural Neuroprotective Agents: Mitigating LPS-Induced Neuroinflammation and Microglial Activation

**DOI:** 10.3390/cells14110780

**Published:** 2025-05-26

**Authors:** Tijana D. Stojanović, Marija R. Rakić, Marija V. Ćosić, Mariana M. Oalđe Pavlović, Aneta D. Sabovljević, Marko S. Sabovljević, Bojan Đ. Božić, Biljana Đ. Božić Nedeljković, Milorad M. Vujičić, Tanja M. Lunić

**Affiliations:** 1 Public Company “Nuclear Facilities of Serbia”, 11000 Belgrade, Serbia; tijana.milovanovic@nuklearniobjekti.rs; 2 Institute of Physiology and Biochemistry “Ivan Djaja”, Faculty of Biology, University of Belgrade, 11000 Belgrade, Serbia; marija.mandic@bio.bg.ac.rs (M.R.R.); bbozic@bio.bg.ac.rs (B.Đ.B.); biljana@bio.bg.ac.rs (B.Đ.B.N.); 3 Institute of Botany and Botanical Garden “Jevremovac”, Faculty of Biology, University of Belgrade, 11000 Belgrade, Serbiamarianao@bio.bg.ac.rs (M.M.O.P.); aneta@bio.bg.ac.rs (A.D.S.); marko@bio.bg.ac.rs (M.S.S.); 4 Department of Plant Biology, Institute of Biology and Ecology, Faculty of Science, Pavol Jozef Šafárik University in Košice, Mánesova 23, 04001 Košice, Slovakia

**Keywords:** bryophytes, moss extract, neuroinflammation, neuroprotection

## Abstract

Neuroinflammation plays a central role in the pathogenesis of neurodegenerative diseases, and there is increasing interest in identifying natural compounds with anti-neuroinflammatory and neuroprotective effects. In this study, we aimed to investigate the biological activities of ethanol and ethyl acetate extracts from five moss species (*Dicranum scoparium*, *Fontinalis antipyretica*, *Hypnum cupressiforme*, *Polytrichum formosum*, and *Tortella tortuosa*) with a focus on their neuroprotective and anti-neuroinflammatory potential. Phytochemical profiling revealed the presence of phenols (up to 24.77 mg GAE/g), phenolic acids (up to 235.48 mg CAE/g), and triterpenoids (up to 367.98 mg UAE/g). A series of *in vitro* assays, including acetylcholinesterase (AChE) and tyrosinase inhibition, MTT, NBT, Griess, and ELISA, were used to assess their bioactivity. Several extracts, particularly ethanolic, significantly inhibited AChE activity, while tyrosinase inhibition was moderate and concentration-dependent. Most extracts maintained >85% cell metabolic activity in BV2 mouse microglia and L929 mouse fibroblasts. Moss extracts significantly suppressed lipopolysaccharide (LPS)-induced production of reactive oxygen species (ROS), nitric oxide (NO), interleukin 6 (IL-6), and tumor necrosis factor alpha (TNF-α) in BV2 cells and reduced microglia-mediated neurotoxicity in undifferentiated SH-SY5Y cells. These findings indicate that moss-derived extracts possess promising anti-neuroinflammatory and neuroprotective properties that warrant further investigation.

## 1. Introduction

Bryophytes, an ancient group of higher plants, are globally distributed and found on every continent. This diverse group comprises three primary divisions: hornworts (Anthocerotophyta), liverworts (Marchantiophyta), and mosses (Bryophyta). They are widespread and adapted to various ecological situations and thus exhibit remarkable diversity. As their ancestors were among the first colonizers of terrestrial ecosystems, bryophytes were frequently exposed to adverse environmental conditions, including attacks by different pathogens, UV radiation, and other stressors [1]. Due to the lack of developed mechanical protection, a high degree of chemical arsenal provides defense mechanisms against unfavorable environmental factors and thus introduces a promising source of biologically highly potent molecules [2].

This plant group has historically received limited attention and was not regarded as a significant source of biologically active compounds [3]. Challenges persist, including the difficulty of accurate identification, the limited natural biomass of bryophytes available for research, their close cohabitation and interaction with other organisms, and their restricted availability in suitable developmental stages. Additionally, the absence of a cuticle makes surface sterilization without damaging the material particularly challenging, which complicates axenic culturing, while field culturing is also out of the question due to low competitiveness with other plants [4]. Despite existing limitations, recent years have seen a growing interest in the chemical composition of bryophytes, which has contributed to the discovery of a large number of biologically active compounds in both mosses [5] and liverworts [6].

Bryophytes have been used in traditional medicine for centuries, with their use dating back to ancient times among tribes in Africa, the Americas, Europe, Australia, Japan, Taiwan, Pakistan, China, Nepal, and various regions of India [7]. They have been utilized for treating burns [8], liver disorders [9], and wound healing [10]. In many cases, scientific evidence has validated their traditional medicinal applications [7,11]. Numerous biomolecules isolated from bryophytes, such as carbohydrates, lipids, proteins, steroids, polyphenols, and terpenoids, have demonstrated antibacterial, antifungal, and antiviral activities [5,7,12]. Moreover, they exhibit other significant properties, such as antitumor activity, inhibition of biochemically significant enzymes, neuroprotective effects, and antioxidant activities [13,14]. While many chemically examined compounds from mosses display remarkable biological activity, particular attention is given to terpenoids and flavonoids. Terpenoids are particularly recognized for their anti-inflammatory and immunomodulatory effects [1], while the antioxidant properties of flavonoids in moss extracts also play a significant role.

Controlled production of reactive oxygen species (ROS) is physiologically essential in humans; however, excessive ROS production without balanced cellular antioxidant responses leads to oxidative stress. Oxidative stress plays a key role in the initiation and progression of various diseases, including cancer [15], cardiovascular conditions [16], ischemia [17], rheumatoid arthritis [18], and the aging process [19]. Oxidative stress can induce neuroinflammation and activate specific signaling pathways in immune cells like microglia [20]. Once activated, microglia release pro-inflammatory cytokines such as tumor necrosis factor-alpha (TNF-α), interleukin-1 beta (IL-1β), and interleukin-6 (IL-6), which amplify the inflammatory response and contribute to neuronal damage [21].

One of the main sources of ROS in immune cells is the enzyme NADPH oxidase (NOX), with the NOX2 isoform playing a particularly important role in microglia [22]. Upon activation, NOX2 catalyzes the formation of superoxide (O_2_^−^), which is subsequently converted into hydrogen peroxide (H_2_O_2_) and hydroxyl radicals (•OH)—highly reactive molecules capable of inflicting direct oxidative damage to neuronal structures [23]. Beyond their cytotoxic effects, these ROS also function as secondary messengers in redox-sensitive signaling pathways that modulate inflammatory responses. In microglia, stimulation with agents such as lipopolysaccharide (LPS) triggers NOX2 activation, leading to excessive ROS production, upregulation of pro-inflammatory cytokines, and the perpetuation of chronic neuroinflammation [24]. This complex interplay between oxidative stress and inflammatory signaling underlies key pathophysiological processes that drive the progressive degeneration of neural tissue. This combination of oxidative stress and neuroinflammation leads to long-term damage and degeneration of neural tissue, aiding in the progression of Alzheimer’s disease [25], Parkinson’s disease [26], depression [27], multiple sclerosis [28], and other neurodegenerative conditions [29]. The biological activities of extracts from certain plant species that contribute to the reduction in neuroinflammation can be investigated using the well-established LPS-induced microglia activation model, which is frequently used in research on neurodegenerative diseases and central nervous system inflammation [30].

Recent studies have shown that various moss species, such as *Dicranum scoparium* Hedw. [31], *Fontinalis antipyretica* Hedw. [32], *Hypnum cupressiforme* Hedw. [33,34,35], *Polytrichum formosum* (Hedw.) G.L. Smith [36], and *Tortella tortuosa* (Schrad. ex Hedw.) Limpr. [37], contain different biologically active compounds, which may make them promising for the treatment of diseases characterized by neurodegeneration and neuroinflammation. In an initial screening, we investigated the antioxidant activity of 20 of the most common moss species in Serbia. Based on these results, we selected the five species that showed the most promising antioxidant potential for further chemical and biological studies. In the present study, the aim was to chemically characterize the ethanol and ethyl acetate extracts of these selected moss species and evaluate their antineurodegenerative potential and neuroinflammatory effects using enzymatic assays and an *in vitro* model of LPS-activated microglial cells. The outcomes could help clarify the mechanisms underlying neuroinflammation and the observed antineurodegenerative effects, potentially opening new avenues for therapeutic interventions in related pathological conditions.

## 2. Materials and Methods

### 2.1. Chemicals

Acetylcholine iodide, acetylcholinesterase from *Electrophorus electricus* Linnaeus, 1766, caffeic acid, DMSO (dimethyl sulfoxide), DTNB (5,5′-dithio-bis(2-nitrobenzoic acid)), Folin–Ciocalteu reagent, galantamine natural for system suitability, gallic acid, L-DOPA (3,4-dihydroxy-l-phenylalanine), ethyl acetate, antibiotics penicillin and streptomycin, RPMI-1640, glucose, LPS (lipopolysaccharide), MTT (3-(4,5-dimethylthiazol-2-yl)-2,5-diphenyltetrazolium bromide), N-(1-naphthyl) ethylenediamine dihydrochloride quercetin, sodium acetate (CH_3_COONa), sodium carbonate anhydrous (Na_2_CO_3_), sodium nitrite (NaNO_2_), sodium phosphate monobasic dihydrate, sulfanilamide, sulfanilic acid, tyrosinase from *Agaricus bisporus* (J.E. Lange) Imbach, and vanillin were obtained from Sigma-Aldrich, St. Louis, MO, USA. Ethanol, glacial acetic acid, and hydrochloric acid were obtained from Zorka Pharma, Šabac, Serbia. Sodium molybdate dihydrate (Na_2_MoO_4_·2H_2_O) was obtained from Dispo-chem, Romsey, UK. Sodium hydroxide (NaOH) was purchased from NRK inženjering, Belgrade, Serbia. Aluminum nitrate nonahydrate (Al(NO_3_)_3_·9H_2_O) and potassium acetate (CH_3_COOK) were obtained from Carlo Erba Reagents, Barcelona, Spain. Methanol and perchloric acid were purchased from VWR, Radnor, PA, USA. FBS (Fetal Bovine Serum) was obtained from GIBCO, Invitrogen, Carlsbad, CA, USA. NBT (Nitro Blue Tetrazolium) was obtained from SERVA, Heidelberg, Germany.

### 2.2. Plant Material

#### 2.2.1. Moss Material Collection

Randomly selected moss material was collected in May 2023 in the wider region of Tara Mountain National Park (West Serbia), stored in paper bags, and left to dry at ambient temperature until application in the experimental procedure. The species include three acrocarpous mosses *Dicranum scoparium*, *Polytrichum formosum*, *and Tortella tortuosa,* and two pleurocarp species *Fontinalis antipyretica* and *Hypnum cupressiforme*. The voucher specimens are kept in the herbarium of the Belgrade University Bryophyte Collection (BEOU-Bryo; s/n).

#### 2.2.2. Preparation of Extracts

A 5 g amount of dried material from each moss species was weighed and powdered using liquid nitrogen. To the ground plant material, 50 mL of a solution of 96% ethanol or ethyl acetate was added. The mixture was then transferred to a glass bottle and incubated for 24 h on an orbital shaker (BioSan PSU-20i, Riga, Latvia) at room temperature. After the incubation, the mixtures were filtered through filter paper, and the clarified extracts were evaporated to dry using a rotary vacuum evaporation apparatus (IKA RV 3 V, IKa-Werke GmbH & Co. KG, Staufen, Germany) set at 40 °C and 800–1000 mbar. They were stored in the dark at 4 °C until use. The yield of the extracts was calculated according to the following formula: [34,38]

Yield (%) = (m1/m2) × 100

In this formula, m1 represents the mass of the dry extract, and m2 corresponds to the mass of the dry plant material used in the extraction [38].

### 2.3. Chemical Characterization

#### 2.3.1. Determination of Total Phenolic Content (TPC)

The determination of TPC was carried out using the spectrophotometric method originally described by Singleton and Rossi [39] with minor modifications. Briefly, 20 μL of the sample extract (1 mg/mL) was mixed with 100 μL of 10% Folin–Ciocalteu reagent and allowed to stand for 6 min at ambient temperature. Afterward, 80 μL of a 7.5% anhydrous sodium carbonate solution was added, and the mixture was incubated in the dark at room temperature for 2 h. Absorbance was read at 740 nm using a Multiskan Sky microplate reader (Thermo Scientific, Vantaa, Finland). A blank sample containing distilled water instead of extract served as the negative control. The TPC was quantified using a gallic acid calibration curve and expressed as mg of gallic acid equivalents per gram of dry extract (mg GAE/g).

#### 2.3.2. Determination of Total Phenolic Acids Content (TPAC)

The quantification of TPAC was performed following the procedure described by Mihailović et al. [40] with slight adjustments. A volume of 10 μL of the extract (1 mg/mL) was mixed with 20 μL of Arnow reagent (containing 10% *w*/*v* sodium molybdate and 10% *w*/*v* sodium nitrite), followed by the addition of 20 μL of 0.1 M HCl and 20 μL of 1 M NaOH. Subsequently, 100 μL of distilled water was added to the reaction mixture, and the absorbance was immediately recorded at 490 nm using a Multiskan Sky microplate reader (Thermo Scientific, Vantaa, Finland). As a control, 50% ethanol was used in place of the extract. TPAC values were calculated using a calibration curve generated from caffeic acid in 50% ethanol, and results were expressed as mg of caffeic acid equivalents per gram of dry extract (mg CAE/g).

#### 2.3.3. Determination of Total Flavonoid Content (TFC)

The TFC of the samples was measured spectrophotometrically according to the procedure given by Park et al. [41]. A reaction mixture was prepared by combining 50 μL of the extract (1 mg/mL) with 205 μL of 80% ethanol, 5 μL of 10% aluminum nitrate nonahydrate, and 5 μL of 1 M potassium acetate. The mixture was then incubated at room temperature for 40 min. After incubation, absorbance was recorded at 415 nm using a Multiskan Sky microplate reader (Thermo Scientific, Vantaa, Finland). For the blank, 96% ethanol was used instead of the extract. TFC was calculated based on a standard calibration curve prepared using quercetin, and results were expressed as milligrams of quercetin equivalents per gram of dry extract (mg QE/g).

#### 2.3.4. Determination of Total Triterpenoid Content (TTC)

The TTC was measured using a previously established method [42]. Briefly, each extract was dissolved in 100% methanol at concentrations of 1 and 10 mg/mL. A 10 µL aliquot from each solution was mixed with 15 µL of a 5% *w*/*v* vanillin-glacial acetic acid solution and 50 µL of perchloric acid. The reaction mixtures were incubated at 60 °C for 45 min and then cooled to room temperature. A control sample containing 100% methanol was prepared in place of the extract. Following the addition of 225 µL of glacial acetic acid, the absorbance was measured at 548 nm using a Multiskan Sky microplate reader (Thermo Scientific, Vantaa, Finland). A calibration curve was constructed using ursolic acid, which was also dissolved in 100% methanol. TTC values were calculated based on the ursolic acid calibration curve and expressed as milligrams of ursolic acid equivalents per gram of dry extract (mg UAE/g dry extract).

### 2.4. Enzyme Assays

#### 2.4.1. Acetylcholinesterase Inhibitory Activity Assay

The determination of AChE inhibitory activity followed a previously described method [43]. This assay assesses the sample’s ability to inhibit AChE, which is pertinent in conditions such as Alzheimer’s disease. The method involves preparing a test reaction mixture consisting of 140 µL of sodium phosphate buffer (0.1 M, pH 7.0), 20 µL of DTNB, 20 µL of sample dissolved in buffer with 5% DMSO (concentrations of 10, 50, 100, 500, and 1000 µg/mL), and 20 µL of AChE solution (5 units/mL) in Tris-HCl buffer (20 mM, pH 7.5). The negative control mixture contained sodium phosphate buffer instead of a sample, while galantamine at the same concentration range as samples acts as the positive control. After a 15 min incubation at 25 °C, the reaction is initiated with acetylcholine iodide, and absorbance is measured at 412 nm using a microtiter plate reader. (Multiskan Sky Thermo Scientific, Vantaa, Finland). The AChE inhibition is calculated using the formula:

Inhibition (%) = [(Ac − As)/Ac] × 100

where Ac is the absorbance of the negative control and As is the absorbance of the sample.

#### 2.4.2. Tyrosinase Inhibitory Activity Assay

The inhibitory activity of Tyr was assessed using a modified method in 96-well plates [44]. The test mixture comprised 80 µL of sodium phosphate buffer (0.1 M, pH 7), 40 µL of Tyr solution (46 units/L), and 40 µL of the sample at concentrations of 10, 50, 100, 500, and 1000 µg/mL. After the addition of 40 µL of L-DOPA in buffer and a 30 min incubation at 25 °C, absorbances were measured at 475 nm using a Multiskan Sky Thermo Scientific, Vantaa, Finland microtiter plate reader. The negative control contained sodium phosphate buffer instead of the sample, while kojic acid at the same concentration range as the samples served as the positive control. Percentage inhibition of Tyr activity was calculated using the formula:

Inhibition (%) = [(Ac − As)/Ac] × 100

where Ac stands for the absorbance of the negative control and As stands for the absorbance of the test sample.

### 2.5. Biological Assays

#### 2.5.1. Cell Cultivation

Mouse fibroblast cell line (L929), cell line originating from mouse microglia (BV2), and cell line derived from SK-N-SH neuroblastoma (undifferentiated SH-SY5Y cells) were used in the present study. All cell lines were obtained from the American Tissue Culture Collection (ATCC, Manassas, VA, USA). The cells were cultured in RPMI-1640 supplemented with 10% FBS, 1% glucose, and 1% antibiotics (penicillin and streptomycin [45,46,47]. They were maintained at 37 °C in a 5% CO_2_ and humidified atmosphere. The final concentrations of the moss extracts were achieved by serial dilution of stock solutions prepared in DMSO. The DMSO concentration was kept constant throughout all experimental conditions and did not exceed 0.1% to avoid solvent-induced cytotoxicity.

#### 2.5.2. BV2 Microglial Cells

Mouse microglial cells (BV2) were seeded in 96-well plates at a density of 1 × 10^4^ cells per well in 100 µL complete medium and incubated for 24 h to allow the cells to attach. After this time, the cells were stimulated with LPS (final concentration 10 µg/mL) and simultaneously treated with moss extracts at final concentrations of 10 or 1 µg/mL. The treatment mixtures were prepared by combining 50 µL of the LPS solution with 50 µL of the moss extract solution so that the final volume was 200 µL per well. The optimal LPS concentration (10 µg/mL) was determined in preliminary experiments by measuring nitric oxide production in response to LPS concentrations ranging from 0.1 to 10 µg/mL, with 10 µg/mL eliciting the highest response. After treatment, BV2 cells were incubated for a further 48 h before being subjected to MTT, NBT, and Griess assays. Non-stimulated cells, cultured in complete medium only, served as controls.

#### 2.5.3. Microglial Culture Supernatant Transfer Model to SH-SY5Y Cells

In this experiment, BV2 microglial cells were stimulated with LPS (final concentration 10 µg/mL) and simultaneously treated with moss extracts (final concentrations 10 and 1 µg/mL). The cells were incubated for 48 h, as described in Section 2.5.2. After 48 h of incubation, the supernatants of these BV2 cultures (100 µL) were collected and transferred to undifferentiated SH-SY5Y cells that had been seeded the previous day at a density of 2 × 10^4^ cells/well in 100 µL of complete medium so that the final volume was 200 µL per well. The SH-SY5Y cells were then incubated with the microglia-conditioned medium for a further 24 h [48]. The metabolic activity of the SH-SY5Y cells was subsequently evaluated using the MTT assay.

#### 2.5.4. Determination of Metabolic Activity (MTT Assay)

This assay measures the reduction in a yellow tetrazolium salt to formazan, a purple crystalline product, facilitated by NAD(P)H-dependent oxidoreductase enzymes found in viable cells [49]. After exposing the cells to the extracts, 100 µL of the cell medium was taken from each well. Subsequently, 10 µL of MTT solution (5 mg/mL) was added to each well, followed by incubation at 37 °C in a 5% CO_2_ environment for 3 h. The formazan crystals formed were solubilized overnight by adding 100 µL of SDS-HCl solution (10% SDS in 0.1 N hydrochloric acid) to each well. Absorbance of the reduced MTT product was subsequently measured at 540/670 nm using a microplate reader (LKB 5060–006, LKB Instruments, Vienna, Austria). Cell metabolic activity was calculated as a percentage by comparing the absorbance values of treated cells to those of the untreated control and multiplying by 100.

#### 2.5.5. Determination of Nitrites Level in Supernatants (Griess Assay)

Nitrite levels were determined using a modified spectrophotometric method originally described by Griess et al. [50]. The Griess reagent, freshly prepared before use, consists of equal volumes of two solutions: N-(1-naphthyl)ethylenediamine dihydrochloride dissolved in distilled water and 1% sulfanilamide (or sulfanilic acid) in 5% phosphoric acid. In this two-step reaction, acidified nitrites produce a nitrosating agent that reacts with sulfanilamide to form a diazonium ion, which then couples with N-(1-naphthyl)ethylenediamine to form a pink azo compound, detectable at 540 nm. After treatment, 50 µL of culture medium from each well was transferred to a clean microplate, followed by the addition of 50 µL of Griess reagent. After 10 min incubation in the dark, the absorbance was measured at 540 nm using a microplate reader (LKB 5060–006, LKB Instruments, Vienna, Austria). Nitrite concentrations were calculated using a sodium nitrite standard curve and expressed as µmol/L (µM).

#### 2.5.6. Determination of Superoxide Anion Radical (NBT Assay)

The concentration of superoxide anion radical (O_2_^−^) in the sample was determined by the Nitro Blue Tetrazolium (NBT) assay [51]. In this assay, NBT is reduced by O_2_^−^ to form diformazan, an insoluble dark blue precipitate. Following incubation of cells with the extracts, the culture medium was partially removed, and NBT solution (5 mg/mL) was added to each well. The plates were then incubated for an additional 3 h at 37 °C in a 5% CO_2_ atmosphere. The resulting formazan crystals were solubilized using SDS-HCl solution. Absorbance of the solubilized product was measured using a microplate reader. The NBT index was calculated as the ratio of absorbance in treated cells to that of the untreated control.

#### 2.5.7. Measurement of Cytokine Levels in Cell Supernatants (ELISA Tests)

Supernatants from LPS-stimulated BV2 cells treated with moss extracts at a final concentration of 10 µg/mL were collected, and the concentrations of cytokines IL-6 (ab222503) and TNF-α (ab208348) were measured. Cytokine quantification was performed using enzyme-linked immunosorbent assay (ELISA) kits, following the manufacturer’s protocol (Abcam, Cambridge, MA, USA). The results are expressed in pg/mL.

#### 2.5.8. Statistical Analysis

Statistical analysis was performed using an independent samples *t*-test (for all cell and enzyme assays) or two-way ANOVA to assess the effects of moss species and solvent type on each chemical parameter, followed by Tukey’s HSD post hoc test in GraphPad Prism, San Diego, CA, USA (version 9.5.1). For all comparisons, *p* < 0.05 for control vs. extract was considered significant. All measurements were performed at least in triplicate, and values are expressed as mean ± standard error.

## 3. Results

### 3.1. Moss Extraction and Chemical Characterization of Extracts

Randomly chosen moss species from inland forests, exposed rocks, and aquatic habitats were the subject of investigation. The yields obtained after moss extraction using ethanol or ethyl acetate are presented in Table 1. Yields from extraction varied from 0.39 to 0.87% for ethyl acetate and from 0.75 to 1.69% for ethanol extracts. A greater extraction yield was observed for ethanol extracts than for ethyl acetate extracts.

The results of total phenolic content (TPC), total phenolic acid content (TPAC), total flavonoid content (TFC), and total triterpenoid content (TTC) of the investigated moss extracts are presented in Table 2.

Phenols were detected in both ethanol and ethyl acetate extracts from all moss species. The highest TPC was recorded in the ethanol extract of *P. formosum* (24.77 mg GAE/g). Ethyl acetate, compared to ethanol, was found to be a more effective solvent for extracting phenolic acids and triterpenoids. The highest TPAC was observed in *H. cupressiforme* (235.48 mg CAE/g), while the highest TTC was measured in *D. scoparium* (367.98 mg UAE/g). Flavonoids were not detected in the extracts of the investigated moss species.

Two-way ANOVA revealed significant interactions between species and solvent for all parameters (*p* < 0.01). Ethanol extracted more TPC than ethyl acetate in *P. formosum* (24.77 ± 0.83 vs. 17.99 ± 0.20 mg GAE/g, *p* = 0.029), while the opposite was true for TPAC in *H. cupressiforme* (235.48 ± 0.00 vs. 20.42 ± 3.43 mg CAE/g, *p* < 0.0001). The ethyl acetate extracts of *D. scoparium* yielded the highest TTC content (367.98 ± 6.19 mg UAE/g), significantly outperforming all other species-solvent combinations (*p* < 0.0001).

### 3.2. Antineurodegenerative Activity

**Figure 1:**
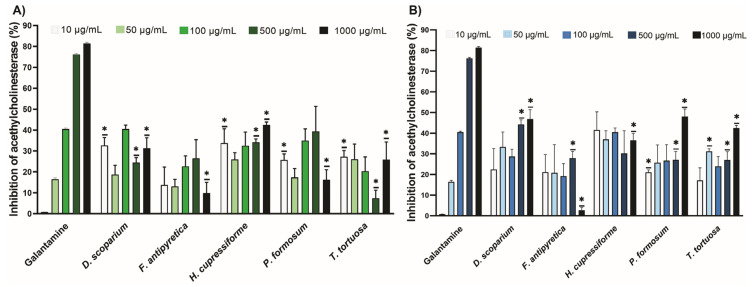
The inhibitory potential of moss ethanol (**A**) and ethyl acetate (**B**) extracts on acetylcholinesterase. All measurements were done in triplicates from one experiment. The results are expressed as the mean ± standard error. Statistical significance was determined using an independent samples *t*-test, comparing each moss extract to the corresponding concentration of galantamine (* *p* < 0.05 extract vs. galantamine at the same concentration).

The results of the inhibitory potential of moss ethanol and ethyl acetate extracts on acetylcholinesterase (AChE) are presented in Figure 1. The AChE inhibitory effect of ethanolic and ethyl acetate extracts at different concentrations is comparable to that of galantamine, which has been used as a standard for enzyme inhibition. The inhibition of AChE was concentration-dependent for the galantamine standard. In contrast, this was not the case for the tested ethanolic and ethyl acetate extracts. The lowest concentration (10 µg/mL) of ethanolic extracts from *D. scoparium*, *H. cupressiforme*, *P. formosum*, and *T. tortuosa* significantly inhibited AChE compared to the standard (Figure 1A). The lowest concentration (10 µg/mL) of ethyl acetate extracts from *P. formosum* significantly inhibited AChE compared to the standard (Figure 1B). At the highest concentrations (1000 µg/mL), both ethanolic and ethyl acetate extracts showed a moderate effect on enzyme inhibition compared to standard galantamine.

**Figure 2:**
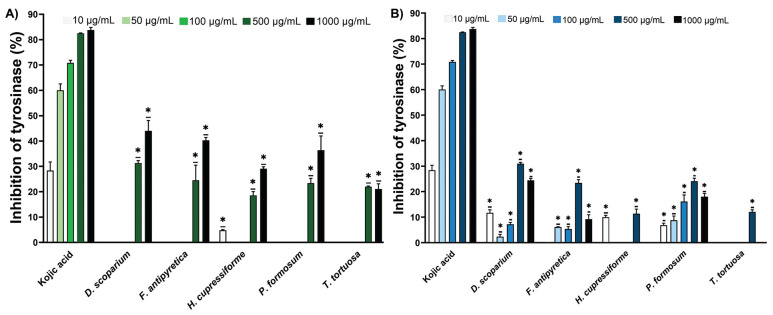
The inhibitory potential of moss ethanol (**A**) and ethyl acetate (**B**) extracts on tyrosinase. All measurements were done in triplicates from one experiment. The results are expressed as the mean ± standard error. Statistical significance was determined using an independent samples *t*-test, comparing each moss extract to the corresponding concentration of kojic acid (* *p* < 0.05 extract vs. kojic acid at the same concentration).

The tyrosinase (Tyr) inhibition data for ethanol and ethyl acetate extracts of moss are shown in Figure 2. Based on the obtained results, the tested extracts did not demonstrate significantly higher Tyr inhibitory activity compared to the reference compound, kojic acid. The ethanolic extracts showed enzyme inhibition mostly at the highest tested concentrations (500 µg/mL and 1000 µg/mL) (Figure 2A). On the other hand, ethyl acetate extracts, at lower concentrations where most of the ethanolic extracts showed no activity, demonstrated a moderate level of enzyme inhibition among the tested concentrations, particularly in *D. scoparium*, *H. cupressiforme*, and *P. formosum* (Figure 2B).

### 3.3. Biocompatibility of Moss Extracts

The biocompatibility of moss extracts was assessed using mouse microglial BV2 cells and L929 mouse fibroblast cells. Following treatment with the extracts, an MTT assay was conducted to evaluate cellular metabolic activity. The results are expressed as percentages relative to the untreated control group, which was assigned a reference value of 100%. Data for BV2 and L929 cells are presented in Table 3 and Table 4, respectively.

The results from Table 3 demonstrate that the overall metabolic activity of BV2 cells treated with all tested extracts at both concentrations (1 µg/mL and 10 µg/mL) remained above 85% relative to untreated control cells (100%), without a significant reduction in metabolic activity. In contrast, metabolic activity in the L929 cells was significantly reduced by several extracts; however, only *F. antipiretica* at the higher concentration tested caused a decrease below 85% compared to the control (Table 4).

### 3.4. Anti-Neuroinflammatory Potential of Moss Extracts

**Figure 3:**
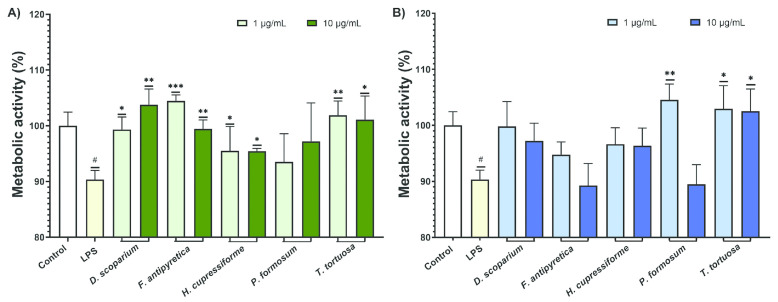
The effects of moss ethanol (**A**) and ethyl acetate (**B**) extracts on metabolic activity of LPS-stimulated BV2 microglial cells. All measurements were done in quadruplicates from three independent experiments, and a representative experiment is shown. The results are presented as the mean ± standard error. Statistical analysis was conducted using an independent samples *t*-test, comparing unstimulated control vs. LPS-stimulated cells (# *p* < 0.05) and LPS-stimulated vs. moss-treated LPS-stimulated cells (* *p* < 0.05, ** *p* < 0.01, *** *p* < 0.001).

The effects of the investigated moss extracts on the metabolic activity of LPS-stimulated BV2 microglial cells were evaluated, and the results are shown in Figure 3.

The results from Figure 3 showed that LPS significantly decreased the metabolic activity of BV2 cells compared to the control group. Most cells treated with ethanolic extracts showed a significant enhancement in metabolic activity compared to the LPS-stimulated group (Figure 3A). In the case of ethyl acetate, a significant increase was observed after treatment with *T. tortuosa* (both concentrations) and *P. formosum* extracts at the lower tested concentration (Figure 3B).

#### 3.4.1. Effects of Moss Extracts on Nitric Oxide and Reactive Oxygen Species Production

**Figure 4:**
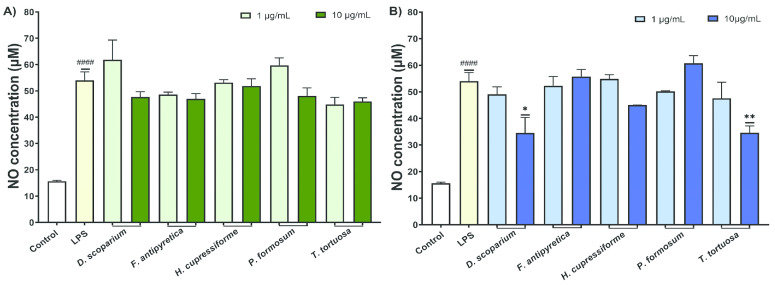
The effects of moss ethanol (**A**) and ethyl acetate (**B**) extracts on nitric oxide production by LPS-stimulated BV2 microglial cells. All measurements were done in quadruplicates from three independent experiments, and a representative experiment is shown. The results are presented as the mean ± standard error. Statistical analysis was conducted using an independent samples *t*-test, comparing unstimulated control vs. LPS-stimulated cells (#### *p* < 0.0001) and LPS-stimulated vs. moss-treated LPS-stimulated cells (* *p* < 0.05, ** *p* < 0.01).

The effects of moss extracts on LPS-stimulated BV2 microglial cells were also determined by measuring the production of nitric oxide (NO), and the results are presented in Figure 4.

The results from Figure 4 demonstrate that activation of untreated control cells with LPS significantly increased the NO production by BV2 cells. Ethanol extracts exhibited weak to moderate activity in reducing NO production, with no statistically significant results (Figure 4A). Conversely, the ethyl acetate extracts from *D. scoparium* and *T. tortuosa* at elevated concentrations significantly reduced NO production in BV2 cells, with statistically significant results (Figure 4B).

**Figure 5:**
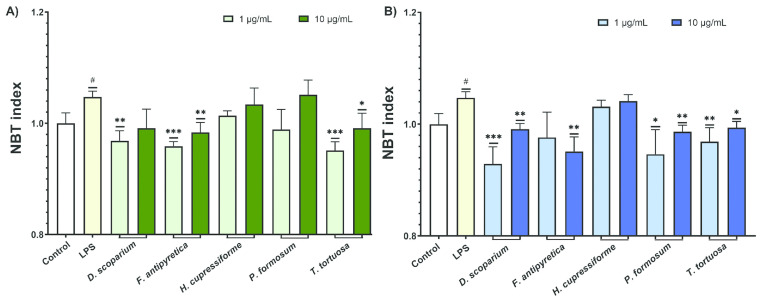
The effects of moss ethanol (**A**) and ethyl acetate (**B**) extracts on reactive oxygen species production by LPS-stimulated BV2 microglial cells. All measurements were done in quadruplicates from three independent experiments, and a representative experiment is shown. The results are presented as the mean ± standard error. Statistical analysis was conducted using an independent samples *t*-test, comparing unstimulated control vs. LPS-stimulated cells (# *p* < 0.05) and LPS-stimulated vs. moss-treated LPS-stimulated cells (* *p* < 0.05, ** *p* < 0.01, *** *p* < 0.001).

The production of ROS was assessed to determine the effects of moss extracts on LPS-stimulated BV2 microglial cells, with data shown in Figure 5.

The results from Figure 5 show that LPS activation significantly increased ROS production in BV2 microglial cells. Some extracts were able to significantly decrease ROS production in these LPS-stimulated cells, highlighting their potential to modulate ROS levels in inflamed microglia. Ethyl acetate extracts exhibited slightly greater activity compared to ethanol extracts. Among the ethanol extracts, those from *F. antipyretica* and *T. tortuosa* at lower concentrations significantly reduced ROS production (Figure 5A). Similarly, ethyl acetate extracts from *D. scoparium*, *P. formosum,* and *T. tortuosa* at both tested concentrations induced a statistically significant decrease in ROS levels (Figure 5B).

#### 3.4.2. Effects of Moss Extracts on the Microglia-Mediated LPS Neurotoxicity

**Figure 6:**
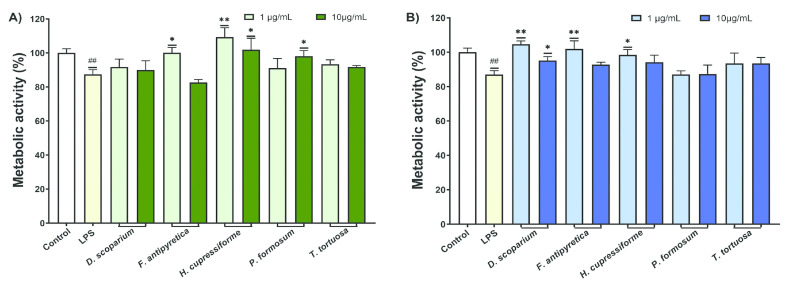
The effects of LPS-stimulated and moss extract ethanol- (**A**) and ethyl acetate-treated (**B**) supernatants of BV2 cells on SH-SY5Y metabolic activity. All measurements were done in quadruplicates from three independent experiments, and a representative experiment is shown. The results are presented as the mean ± standard error. Statistical analysis was conducted using an independent samples *t*-test, comparing unstimulated control vs. LPS-stimulated cells (## *p* < 0.01) and LPS-stimulated vs. moss-treated LPS-stimulated cells (* *p* < 0.05, ** *p* < 0.01).

To investigate whether investigated moss extracts can provide protection to undifferentiated SH-SY5Y cells against neurotoxicity induced by LPS, the metabolic activity of SH-SY5Y cells was measured. The effects of BV2 supernatants treated with LPS and moss extracts on SH-SY5Y cells are presented in Figure 6.

The results from Figure 6 indicate that supernatants from BV2 cells treated only with LPS significantly reduced the metabolic activity of SH-SY5Y cells. When SH-SY5Y cells were treated with supernatants from BV2 cells that were simultaneously treated with LPS and moss extracts, there was an increase in metabolic activity. The ethanolic extracts of *H. cupressiforme* showed the best results in both tested concentrations, as they significantly increased the metabolic activity of the cells compared to the control. In addition, *F. antipyretica* showed significant results at lower concentrations, while *P. formosum* showed significant effects at higher concentrations (Figure 6A). In the case of ethyl acetate, the best activity was observed in *F. antipyretica* and *H. cupressiforme* at lower concentrations, while *D. scoparium* showed a significant increase in cell metabolic activity at both tested concentrations (Figure 6B). These results suggest that some of the moss extracts tested could provide protection against microglia-mediated LPS neurotoxicity.

#### 3.4.3. Effects of Moss Extracts on Tumor Necrosis Factor Alpha and Interleukin-6 Production

**Figure 7:**
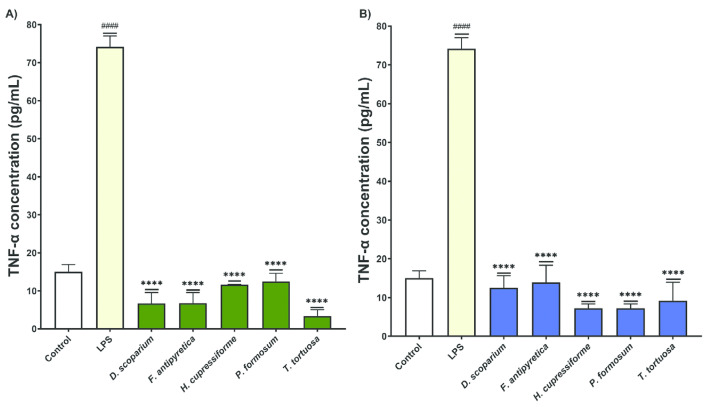
The effects of moss ethanol (**A**) and ethyl acetate (**B**) extracts on tumor necrosis factor alpha production by LPS-stimulated BV2 microglial cells. All measurements were done in triplicates from one experiment. The results are presented as the mean ± standard error. Statistical analysis was conducted using an independent samples *t*-test, comparing unstimulated control vs. LPS-stimulated cells (#### *p* < 0.0001) and LPS-stimulated vs. moss-treated LPS-stimulated cells (**** *p* < 0.0001).

The effects of moss extracts on LPS-stimulated BV2 microglial cells were determined by measuring the production of tumor necrosis factor alpha (TNF-α), and the results are presented in Figure 7.

Microglial cells treated with LPS alone show a significant increase in TNF-α production compared to the control group. This confirms that LPS induces an inflammatory response in BV2 cells. Following treatment with both ethanol and ethyl acetate moss extracts, there is a significant reduction in TNF-α production by BV2 cells compared to the LPS-only group. These results suggest that the moss extracts exhibit anti-inflammatory properties, effectively reducing the production of TNF-α. All extracts (both ethanol and ethyl acetate) significantly decreased TNF-α production by BV2 cells compared to the LPS-treated group (Figure 7).

**Figure 8:**
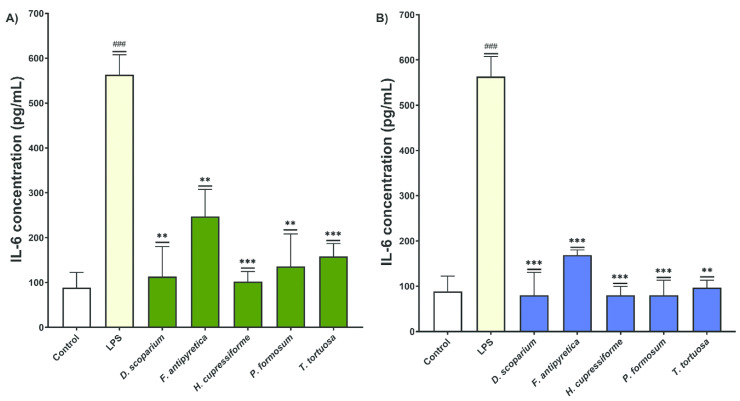
The effects of moss ethanol (**A**) and ethyl acetate (**B**) extracts on interleukin-6 production by LPS-stimulated BV2 microglial cells. All measurements were done in triplicates from one experiment. The results are presented as the mean ± standard error. Statistical analysis was conducted using an independent samples *t*-test, comparing unstimulated control vs. LPS-stimulated cells (### *p* < 0.001) and LPS-stimulated vs. moss-treated LPS-stimulated cells (** *p* < 0.01, *** *p* < 0.001).

The effects of moss extracts on LPS-stimulated BV2 microglial cells were also determined by measuring the production of interleukin-6 (IL-6), and the results are presented in Figure 8.

The cells treated with LPS alone showed a significant increase in IL-6 production in the supernatants of BV2 cells compared to the control group, confirming that LPS induces an inflammatory response in BV2 cells. Treatment with both ethanol and ethyl acetate moss extracts resulted in a significant decrease in IL-6 production in BV2 cells compared to the group treated with LPS alone (Figure 8). These results indicate that the tested moss extracts have significant anti-inflammatory effects and influence both TNF-α and IL-6 production, suggesting their therapeutic potential in modulating cytokine responses in inflammatory conditions.

## 4. Discussion

This study investigates the chemical characterization and biological effects against neuroinflammation, neurodegeneration, of ethanolic and ethyl acetate extracts from five moss species collected from Tara Mountain National Park in Serbia. The chemical composition of mosses is attracting increasing interest due to their potential biological activities, but there are still considerable gaps in the literature in relation to specific species. In this study the chemical characterization (TPC, TPAC, TFC, and TTC) was performed spectrophotometrically, showing solvent-dependent extraction patterns strongly influenced by metabolite polarity. Ethanol (polar) excelled for TPC, aligning with phenolic hydroxyl group solubility [52]. Conversely, ethyl acetate (moderately polar) dominated for triterpenoids, consistent with their lipophilicity [53].

Ethyl acetate extracts of *D. scoparium* exhibited a very high TPAC and TTC compared to the ethanol extracts of this moss species. Information on the chemical composition of *D. scoparium* extracts is available, but results vary depending on the solvent used during extraction [54]. It has been shown in the literature that they have low levels of flavonoids but are rich in phenolic acids. In this study, along with high TPAC, higher TPC values were also recorded compared to those reported in the literature [31]. Ethanolic extracts of *F. antipyretica* demonstrated better results than ethyl acetate extracts for all parameters except for TTC. However, only limited data are available for *F. antipyretica*, with studies mainly focusing on the fatty acid composition of the lipids [32]. Ethyl acetate extracts of *H. cupressiforme* exhibited a very high TPAC and TTC. In the *H. cupressiforme* case, 14 phenolic compounds have been previously identified, including kaempferol and major phenolic acids such as p-hydroxybenzoic acid, protocatechuic acid, and caffeic acid, although TPC reported in ethanol extracts was lower than observed in this study [35]. Ethanolic and ethyl acetate extracts of *P. formosum* exhibited high TTC, which is not previously found in the literature. Similarly, although the TPC in *P. formosum* extract has been documented, the reported values were lower than those found herein. Seasonal chemical analysis revealed a diverse composition of sugars and sugar alcohols in this species, emphasizing its metabolic versatility [36]. The ethanolic extracts of *T. tortuosa* exhibited a higher TPC, while the ethyl acetate extracts showed a higher TPAC. Literature data show that for *T. tortuosa*, phenolic components such as salicylic acid, gallic acid, caffeic acid, and genistic acid have been identified using HPLC-TOF/MS, highlighting its significance as a natural source of bioactive agents [37].

Variations in TPC, TPAC, and other secondary metabolite levels between studies can be affected by several factors, including the geographical location of the collection, seasonal factors, and/or the extraction techniques [34,55]. Additionally, differences in experimental conditions such as solvent type, extraction time, temperature, extract concentration, and characterization techniques can significantly impact the measured metabolite levels [33,34]. Nevertheless, the chemical characterization in this study presents novel findings into the composition of the five investigated moss species from Tara Mountain National Park, enhancing our understanding and contributing to the knowledge of their bioactive potential and setting the stage for further research.

The AChE inhibitory potential of investigated moss extracts has been sparsely explored. Literature suggests that phenol- and flavonoid-rich extracts can serve as natural AChE inhibitors, offering potential therapy for cognitive disorders like Alzheimer’s disease [56]. The moss extracts in this study did not show a concentration-dependent inhibition of AChE. This trend has been previously observed with plant-based inhibitors, where higher concentrations can lead to mutual interactions that block or reduce their activity [33]. In addition, lower concentrations may allow optimal occupancy of the AChE receptor without steric hindrance or antagonism. Among the investigated species, only *H. cupressiforme* has been investigated so far, showing higher AChE inhibition at lower concentrations, which is consistent with the results obtained in this study [33]. Based on the phytochemical profile of the extracts and previous docking studies on *H. cupressiforme* [34], the inhibition is likely mediated by secondary metabolites that bind to the AChE active site and prevent substrate access. These compounds have been shown to interact with residues essential for enzymatic activity, including those of the catalytic triad, such as Ser200 and His440. For other investigated moss species, there are no previous data on AChE inhibitory potential, making this study the first to report their moderate activity and positioning these mosses as putative new sources of natural AChE inhibitors.

Inhibition of Tyr, an important target in neurodegenerative diseases, has been associated with phenol- and flavonoid-rich plants [57]. In this study, ethanolic extracts showed a clear dose-dependent inhibition, while ethyl acetate extracts showed higher activity at lower concentrations and did not show a strict dose-response trend. This aligns with previous findings and may be attributed to differences in the solubility and interactions of bioactive compounds across concentrations and solvents [33,34]. Among the studied species, only *H. cupressiforme* has been previously investigated for Tyr inhibition, showing moderate to high activity in ethanolic and ethyl acetate extracts higher than observed in this study [33]. This discrepancy could be due to differences in geographical origin and extraction methods, as the earlier study [33] used a Soxhlet extraction, whereas this study used maceration. For the remaining species, this is the first report of their Tyr inhibitory potential. If the research were to continue, these moss species might have the potential as natural sources of Tyr inhibition.

To further investigate the observed antineurodegenerative potential of moss extracts, their effects were studied in a well-established LPS-activated microglia model, which is known for its relevance in the study of neuroinflammation and neurodegenerative diseases [29,30]. As oxidative stress and neuroinflammation are critical factors in the advancement of these diseases, LPS-induced microglia activation functions as an important model for evaluating the anti-inflammatory and antioxidant properties of moss extracts. An important aspect in the evaluation of the anti-neuroinflammatory and antineurodegenerative potential of these extracts is the ability of their secondary metabolites to cross the blood-brain barrier (BBB). The literature indicates that several natural compounds, including phenolic acids and flavonoids found in mosses, can indeed penetrate the BBB and exert effects in the CNS [58,59,60].

The results of this study show that some of the moss extracts tested significantly reduce inflammatory mediators (ROS and NO) and proinflammatory cytokines (TNF-α and IL-6) in LPS-activated microglia. Of the samples tested, the extracts of *D. scoparium*, *F. antipyretica,* and *T. tortuosa* were the most effective in lowering ROS levels (Figure 5), while only the ethyl acetate fractions of *D. scoparium* and *T. tortuosa,* and only at the higher concentration tested (10 µg/mL), significantly reduced NO production (Figure 4). This divergence between the inhibition of NO and ROS likely reflects their regulation by different inflammatory pathways: NO synthesis relies on the transcriptional upregulation of inducible nitric oxide synthase (iNOS) via NF-*κ*B activation, whereas ROS, especially superoxide anions, are rapidly generated by NOX2 activation, which is controlled by post-translational events and additional signaling molecules such as PKC and Rac1 [61,62]. The stronger suppression of ROS therefore suggests that certain extracts preferentially target NOX2-mediated oxidative processes. In addition, the choice of solvent clearly influences activity: ethyl acetate extracts of *D. scoparium* and *T. tortuosa* significantly decreased NO levels, whereas their ethanol extracts did not, consistent with reports that the extraction solvent may differentially affect ROS and NO inhibition [63]. Finally, many polyphenols, although potent ROS scavengers, do not uniformly inhibit NO; for example, rutin and cyanidin-3-glucoside suppress ROS but have little effect on NO production [63]. Taken together, these results emphasize the importance of both the extraction method and the specific phytochemical profiles in determining anti-inflammatory efficacy. In addition, all tested extracts successfully reduced IL-6 and TNF-α levels, significant neuroinflammatory mediators, to control cell levels. The levels of IL-6 and TNF-α were reduced by 87–88% following treatment with ethanol extracts and by 62–65% after treatment with ethyl acetate extracts, depending on the moss species.

The neuroprotective effects were further confirmed in a supernatant transfer model, where the reduction in SH-SY5Y cell metabolic activity was not caused by direct LPS exposure but occurred indirectly through cytotoxic mediators released by the activated microglia. Upon LPS stimulation, microglial cells release ROS, NO, and pro-inflammatory cytokines, which can induce oxidative stress, mitochondrial dysfunction, and inflammation in surrounding neurons, ultimately leading to decreased SH-SY5Y metabolic activity. This indirect mechanism of neurotoxicity has also been reported in several other studies [34,48]. In particular, extracts from *D. scoparium*, *F. antipyretica,* and *H. cupressiforme* were particularly effective, suggesting their potential neuroprotective properties. While all species showed similar efficacy in reducing IL-6 and TNF-α levels, some species, like *D. scoparium*, also significantly reduced ROS and NO production, highlighting its dual potential as an anti-inflammatory and neuroprotective agent.

Previous research has investigated *H. cupressiforme*, while studies on the effects of other moss species in similar cell models for neuroinflammation are lacking. Earlier studies showed that extracts of *H. cupressiforme* reduce the production of NO, ROS, and proinflammatory cytokine [33,34], providing a basis for studying similar effects in other moss species. In comparison with the previous study [34], which reported a significant decrease in IL-6 levels in cells treated with *H. cupressiforme* compared to LPS-treated controls, TNF-α production was not significantly reduced by *H. cupressiforme*. In contrast, this study observed a statistically significant reduction in both cytokine levels. Variations in the results could be explained by differences in extraction methods and the geographical origin of the mosses.

Finally, this study provides, to the best of our knowledge, the first evidence of inhibition of ROS, NO, and cytokine production (IL-6 and TNF-α) and protection against LPS-induced neurotoxicity by extracts of *D. scoparium*, *F. antipyretica*, *P. formosum,* and *T. tortuosa*. These results open up new possibilities for their therapeutic application. This research not only builds on previous studies but also fills important gaps in the literature regarding the bioactivity of investigated moss species and highlights their potential as an important provider of anti-inflammatory and neuroprotective substances.

## 5. Conclusions

In this study, ethanol and ethyl acetate extracts of five mosses: *D. scoparium, F. antipiretica, H. cupressiforme, P. formosum,* and *T. tortuosa* from the Tara Mountain National Park in Serbia were investigated for their antineurodegenerative, anti-inflammatory, and neuroprotective properties. The chemical characterization of the extract uncovered significant levels of phenols, phenolic acids, and triterpenoids, all of which are secondary metabolites known for their notable biological activity. Moss extracts demonstrated the potential to alleviate oxidative and inflammatory stress in LPS-activated BV2 microglial cells by inhibiting the production of inflammatory mediators, including ROS, NO, IL-6, and TNF-α. The extracts also reduced the neurotoxic effects of activated microglia on surrounding cells (SH-SY5Y). This evidence for the anti-neuroinflammatory and neuroprotective properties of the moss was further validated by evaluating its inhibitory potential against AChE, with the extracts exhibiting higher inhibition rates at low concentrations compared to the corresponding standard compounds. These experiments, including the evaluation of Tyr inhibitory activity, were performed for the first time with the species *D. scoparium, F. antipiretica, P. formosum,* and *T. tortuosa*, making this research a pioneering work in the evaluation of their anti-inflammatory and neuroprotective properties. Overall, the results of this study highlight the potential of moss extracts as novel anti-inflammatory agents, warranting further investigation of their mechanisms of action and potential clinical applications.

## Figures and Tables

**Table 1:** Extraction yield for moss ethanol and ethyl acetate extracts.

Moss Species	Extraction Yield (%)	
	Ethanol Extracts	Ethyl Acetate Extracts
*Dicranum scoparium*	1.69	0.87
*Fontinalis antipyretica*	0.99	0.82
*Hypnum cupressiforme*	0.75	0.39
*Polytrichum formosum*	1.61	0.71
*Tortella tortuosa*	1.69	0.61

**Table 2:** Chemical characterization of moss ethanol and ethyl acetate extracts.

Moss Species	TPC (mg GAE/g Extract)		TPAC (mg CAE/g Extract)		TFC (mg QE/g Extract)		TTC (mg UAE/g Extract)	
	Ethanol	Ethyl Acetate	Ethanol	Ethyl Acetate	Ethanol	Ethyl Acetate	Ethanol	Ethyl Acetate
*D. scoparium*	10.02 ± 0.08 ^a^	10.52 ± 0.92 ^a^	11.39 ± 2.01 ^a^	188.69 ± 2.46 ^B^	ND	ND	120.66 ± 3.22 ^a^	367.98 ± 6.19 ^D^
*F. antipyretica*	13.94 ± 0.60 ^b^	4.94 ± 0.10 ^a^	23.70 ± 2.84 ^a^	ND	ND	ND	105.21 ± 3.22 ^a^	123.44 ± 8.61 ^B^
*H. cupressiforme*	5.60 ± 0.73 ^a^	14.77 ± 1.71 ^b^	20.42 ± 3.43 ^a^	235.48 ± 0.00 ^C^	ND	ND	89.77 ± 0.64 ^a^	260.30 ± 5.15 ^C^
*P. formosum*	24.77 ± 0.83 ^c^	17.99 ± 0.20 ^b^	55.71 ± 2.01 ^b^	122.20 ± 4.02 ^A^	ND	ND	130.31 ± 5.15 ^a^	220.40 ± 11.58 ^B^
*T. tortuosa*	19.10 ± 0.75 ^b^	3.33 ± 1.41 ^a^	ND	156.68 ± 4.02 ^B^	ND	ND	150.90 ± 5.94 ^a^	259.87 ± 7.48 ^C^

CAE—caffeic acid equivalents, GAE—gallic acid equivalents, ND—not detected, QE—quercetin equivalents, TFC—total flavonoid content, TPC—total phenolic content, TPAC—total phenolic acid content, TTC—total triterpenoid content, UAE—ursolic acid equivalents. All measurements were done in triplicates from one experiment. The results are expressed as the mean ± standard error. Statistical analysis was performed using two-way ANOVA to assess the effects of solvent and moss species on the chemical contents. Different superscript letters indicate significant differences (Tukey’s test, *p* < 0.05) between solvents (lowercase, within species) or species (uppercase, within solvent).

**Table 3:** Biocompatibility testing of moss extracts on BV2 cells. All measurements were done in quadruplicates from three independent experiments, and a representative experiment is shown. The results are expressed as the mean ± standard error. Statistical significance was assessed using an independent samples *t*-test, comparing each treatment group to the corresponding untreated control.

Moss Species	Metabolic Activity (%)			
	Ethanol Extracts		Ethyl Acetate Extracts	
	1 µg/mL	10 µg/mL	1 µg/mL	10 µg/mL
Control	100 ± 3.91			
*D. scoparium*	96.96 ± 1.63	93.14 ± 2.14	96.96 ± 1.66	91.09 ± 2.89
*F. antipiretica*	99.34 ± 0.86	98.24 ± 3.39	95.69 ± 3.88	85.28 ± 3.29
*H. cupressiforme*	91.53 ± 1.99	88.67 ± 4.15	94.84 ± 2.44	104.05 ± 2.94
*P. formosum*	112.47 ± 1.69	101.31 ± 1.93	98.04 ± 0.29	91.63 ± 2.86
*T. tortuosa*	92.98 ± 4.40	90.9 ± 0.87	108.34 ± 1.99	100.78 ± 3.32

**Table 4:** Biocompatibility testing of moss extracts on L929 cells. All measurements were done in quadruplicates from three independent experiments, and a representative experiment is shown. The results are expressed as the mean ± standard error. Statistical significance was assessed using an independent samples *t*-test, comparing each treatment group to the corresponding untreated control (* *p* < 0.05, ** *p* < 0.01, *** *p* < 0.001).

Moss Species	Metabolic Activity (%)			
	Ethanol Extracts		Ethyl Acetate Extracts	
	1 µg/mL	10 µg/mL	1 µg/mL	10 µg/mL
Control	100 ± 2.32			
*D. scoparium*	87.7 ± 1.11 **	88.45 ± 2.14 **	91.30 ± 1.92 *	92.25 ± 1.95
*F. antipiretica*	94.08 ± 1.30	90.82 ± 1.66 *	91.64 ± 3.27	77.35 ± 1.08 ***
*H. cupressiforme*	95.18 ± 4.47	92.18 ± 1.25 *	85.48 ± 0.84 **	94.40 ± 2.58
*P. formosum*	101.38 ± 3.01	90.55 ± 2.39 *	97.66 ± 4.41	86.07 ± 1.35 **
*T. tortuosa*	95.59 ± 3.87	101.16 ± 4.17	91.71 ± 2.97	91.89 ± 1.69 *

## Data Availability

Data are contained within the article.

## Abbreviations

The following abbreviations are used in this manuscript:

AChE	Acetylcholinesterase
ATCC	American Tissue Culture Collection
BBB	blood-brain barrier
BV2	Mouse microglial cell line
CAE	Caffeic acid equivalents
DMSO	Dimethyl sulfoxide
DTNB	5,5′-Dithio-bis(2-nitrobenzoic acid)
ELISA	Enzyme-linked immunosorbent assay
FBS	Fetal bovine serum
GAE	Gallic acid equivalents
IL-6	Interleukin-6
L-DOPA	3,4-Dihydroxy-L-phenylalanine
LPS	Lipopolysaccharide
MTT	3-(4,5-Dimethylthiazol-2-yl)-2,5-diphenyltetrazolium bromide
NBT	Nitro blue tetrazolium
NO	Nitric oxide
QE	Quercetin equivalents
ROS	Reactive oxygen species
RPMI	Roswell Park Memorial Institute medium
SH-SY5Y	Human neuroblastoma cell line
TFC	Total flavonoid content
TNF-α	Tumor necrosis factor alpha
TPC	Total phenolic content
TPAC	Total phenolic acid content
TTC	Total triterpenoid content
Tyr	Tyrosinase
UAE	Ursolic acid equivalents
